# Morphometric mapping of ACL femoral footprint variability: implications for personalized anatomic single-bundle reconstruction

**DOI:** 10.3389/fsurg.2026.1897000

**Published:** 2026-10-21

**Authors:** Dongliang Zhang, Zhongbao Han, Yinxiu Chi

**Affiliations:** Jiangsu Medical College, Yancheng, Jiangsu, China

**Keywords:** anterior cruciate ligament, femoral footprint, morphometric analysis, personalized reconstruction, precision medicine

## Abstract

**Background:**

While the Ribbon Theory has challenged traditional two-bundle anatomy, the clinical translation of ACL morphological variability remains unclear. The field is evolving from “anatomic” toward “Precision Anatomic Reconstruction,”which motivates the development of quantitative criteria for individualized surgical planning.

**Methods:**

Thirty fresh-frozen cadaveric knees (15male, 15female) underwent standardized low-temperature perfusion preservation and comprehensive 3D morphometric analysis. Correlations between footprint characteristics and anthropometric parameters were evaluated.

**Results:**

Ribbon-like morphology predominated (80%), yet substantial inter-individual variability existed in footprint morphology:oval (66.7%), teardrop (23.3%), and irregular (10.0%).Males demonstrated significantly larger footprint area than females (144 ± 19 vs. 124 ± 18 mm^2^, *p* < 0.01). Critical finding: Footprint area correlated strongly with ACL width (r = 0.72, *p* < 0.001), but not with height or weight. Conventional round tunnels (9 mm) provided only 47.5% footprint coverage, creating a significant “coverage gap.”

**Conclusions:**

ACL morphology exhibits high heterogeneity that cannot be predicted by anthropometric measures. This study advocates for a paradigm shift towards Precision Anatomic Anterior Cruciate Ligament (ACL) Reconstruction, emphasizing that oval large footprints may suggest consideration of elliptical tunnels and rectangular grafts. Additionally, it identifies that true two-bundle phenotypes, comprising 13.3% of cases, along with intermediate phenotypes, accounting for 6.7% (a combined total of 20%), may still benefit from the consideration of double-bundle techniques. For the small subgroup of irregular footprints (10%, *n* = 3), anatomical observations from this limited-size cohort raise the hypothetical possibility of using three-dimensional-printed patient-specific guides and AI-assisted preoperative planning. Further validation in larger study populations is required before clinical application.

## 1 Introduction

Anterior cruciate ligament (ACL) reconstruction represents one of the most commonly performed orthopedic procedures, with increasing incidence globally (1, 2). Despite advancements in technology, residual rotational instability and the early onset of osteoarthritis continue to pose significant challenges, affecting 15%–25% of patients following conventional reconstruction methods (3, 4).

Traditionally, it has been believed that the anterior cruciate ligament (ACL) consists of two functionally distinct bundles: the anteromedial (AM) and posterolateral (PL) bundles (5). However, recent long-term clinical trials and meta-analyses have shown that anatomic single-bundle (ASB) and double-bundle reconstructions yield comparable functional outcomes over a 5–10 year follow-up period (6, 7). This suggests that the precision of graft placement in relation to the native footprint may be more crucial than the number of bundles used (8).

Additionally, the Ribbon Theory has fundamentally challenged traditional anatomical models. Groundbreaking research has revealed that the ACL is primarily a single, flat, ribbon-like structure with a broad femoral attachment (9). This anatomical understanding has spurred the development of anatomic single-bundle techniques that focus on the complete restoration of the footprint (10, 11).

Despite recent advancements, significant translational challenges remain. The Ribbon Theory provides a general description of ACL morphology but fails to account for the considerable inter-individual variability in footprint dimensions observed in clinical settings. Current surgical practices often utilize standardized tunnel diameters (8–10 mm), which may lead to anatomical mismatches (12, 13).

Recent biomechanical studies suggest that the percentage of footprint coverage, rather than the absolute size of the graft, is associated with the restoration of rotational stability (14). Additionally, a 2024 editorial preface on “Precision Anterior Cruciate Ligament Reconstruction” emphasized the clinical importance of preoperative morphometric assessment for personalized ACL surgery (15). However, there are no established quantitative criteria that link specific anatomical phenotypes to optimal surgical techniques.

This study aims to address these critical gaps by conducting a comprehensive morphometric analysis of ACL anatomical variability.

## 2 Materials and methods

### 2.1 Ethical approval and specimen procurement

This anatomical morphometric study utilized human cadavers voluntarily donated through the institutional body-donation program. All cadavers were acquired with prior written pre-mortem consent for anatomical research purposes. The study received approval from the institutional ethics committee at Jiangsu Medical College. As de-identified cadaveric specimens were employed, additional patient-level informed consent was deemed unnecessary for this investigation. No experiments involving animals or living human participants were conducted. The study was approved by the Institutional Review Board. Thirty fresh-frozen cadaveric knees (mean age 62.4 ± 14.2 years) were procured from the institutional body donation program between January 2021 and December 2024, with written informed consent. Thirty knees (comprising 15 pairs of right and left knees) were obtained from 15 fresh-frozen human cadaveric donors. Paired-samples t-tests were conducted to compare measurements between the right and left knees from the same cadaver. The exclusion criteria included a history of knee surgery or trauma, advanced arthritis (Kellgren-Lawrence grade ≥3), age under 18 years, and systemic connective tissue diseases. Male cadavers were significantly taller and heavier than female cadavers, with *p*-values of <0.001 and <0.01, respectively. All cadaver specimens were preserved using standard femoral artery perfusion. This preservation method maintains physiological tissue turgor by ensuring consistent interstitial hydration and preventing desiccation, shrinkage, or morphological distortion of periarticular soft tissues and ligament insertions. This widely adopted technique for preserving knee specimens in cadaveric studies has been validated to stabilize the native morphology and dimensional characteristics of ligaments without causing significant tissue stretching or contraction, thereby ensuring accurate morphometric measurements of the anterior cruciate ligament (ACL) footprint.

### 2.2 Specimen preservation protocol

To maintain the integrity of native tissue architecture and mitigate shrinkage artifacts commonly associated with traditional formalin fixation, we implemented a standardized low-temperature perfusion preservation protocol conducted at 4 °C. Perfusion was executed using a closed extracorporeal constant-flow perfusion model (Figure 1A). The stock perfusate formulation was tailored for cadaveric specimens with a reference body weight of 50 kg, comprising 1,000 mL of 10% formaldehyde, 3,000 mL of 75% ethanol, and 500 mL of ammonia water, supplemented with 5,500 mL of purified water to achieve a working solution with a total volume of 10,000 mL. The cadaver was positioned supine, and an 8-cm longitudinal incision was made at the midpoint of the inguinal region to expose the common femoral artery (Figure 1B). The pump-head speed was set to 0.6–1.2 mL min^−1^, approximately 1% of the physiological flow rate of the common femoral artery (60–120 mL min^−1^), with a total perfusion duration of 12 h to minimize mechanical injury to the vascular endothelium.

**Figure 1:**
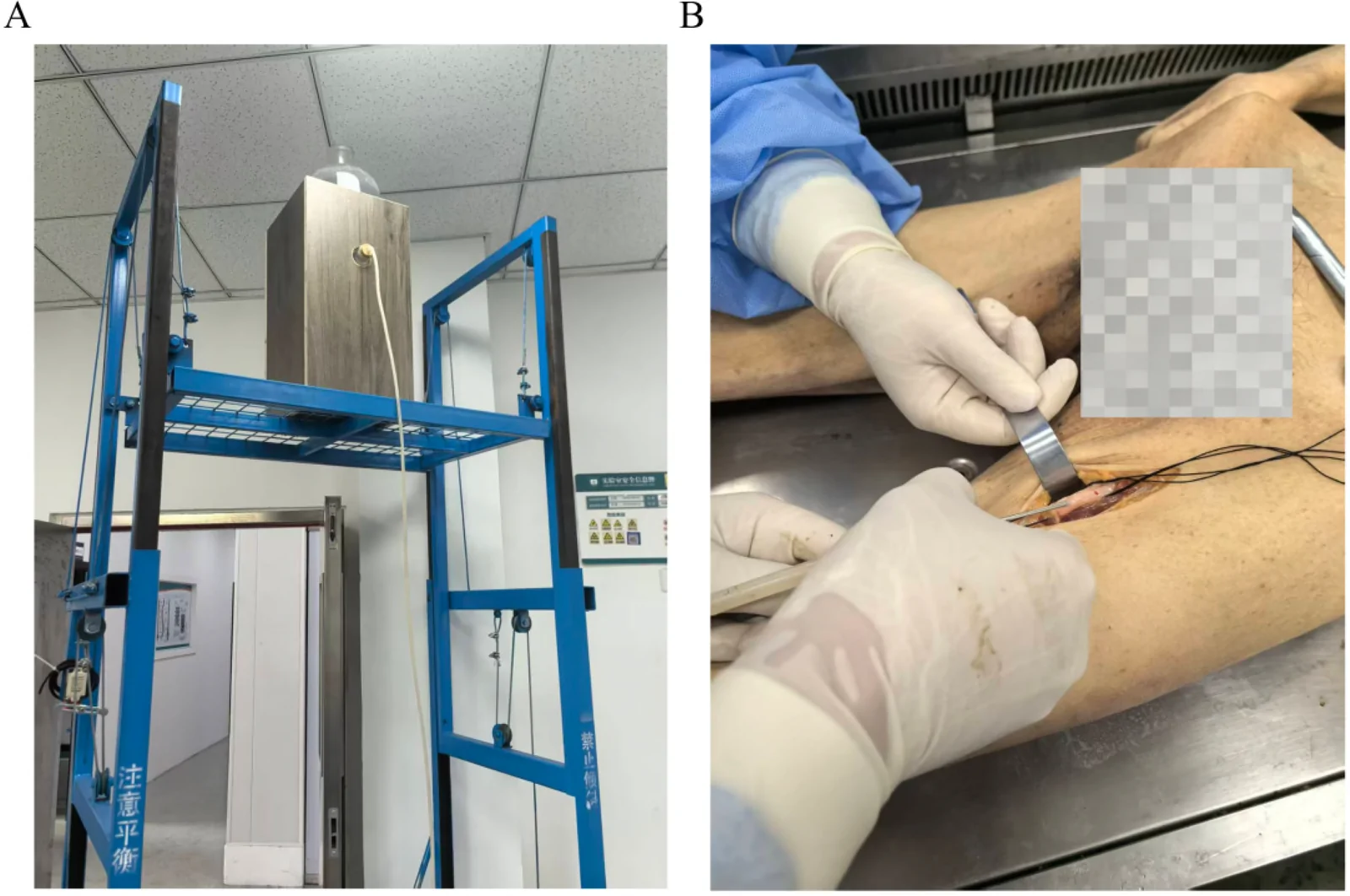
Cadaver femoral artery perfusion procedure for specimen preservation. **(A)** Self-made electric infusion apparatus used for perfusing cadaveric specimens. **(B)** Puncture operation performed on the surgically exposed femoral artery for delivery of preservative solution.

### 2.3 Dissection and morphological assessment

Each specimen was positioned with the knee flexed at 90 degrees. Following an anteromedial arthrotomy, the morphology of the ACL was classified according to the criteria established: Type R (Ribbon—single flat structure), Type B (Two-bundle-distinct anteromedial/posterolateral separation), or Type I (Intermediate-partial definition). After detachment from the tibia while preserving the femoral footprint, the total length of the ACL was measured using digital calipers with an accuracy of 0.01 mm (Mitutoyo), and the mid-substance width was determined using a standardized grid technique. The boundary of the femoral ACL footprint was meticulously defined as the entire macroscopic area of ligament insertion, including the peripheral fiber attachment zone, which was visualized following careful debridement of soft tissue. Standardized photographs were taken perpendicular to the plane of the femoral notch using a fixed-distance rig, with a built-in scale ruler included in each frame for spatial calibration. Prior to planimetric analysis, image calibration was conducted in ImageJ by using the physical scale marker to convert pixel measurements to millimeters. The footprint area was determined by manually tracing the defined insertion border in ImageJ. An elliptical approximation was derived by fitting an ellipse to the delineated boundary of the footprint, with the major and minor axes automatically computed to assess morphological elongation. All morphological measurements were independently conducted by two observers, and the final dataset was compiled using the mean values of these independent measurements across all specimens.

### 2.4 Morphological classification criteria

The morphological classification of the femoral footprint was conducted by integrating quantitative morphometric indices with macroscopic visual inspection. The classification criteria were as follows: 1. Oval footprint: Characterized by a regular elliptical contour, and a symmetric outline devoid of significant constriction or protrusion. 2. Teardrop footprint: Defined by a unilateral tapering narrowing at one pole, and pronounced unilateral asymmetry of the insertion margin. 3. Irregular footprint: Exhibited a non-elliptical, jagged contour with a heterogeneous margin, which could not be accurately approximated by a simple oval or teardrop shape. Further categorization of ligament midsubstance morphology was performed as follows: 1. Two-bundle phenotype: Displayed a macroscopically distinct separation into well-defined anteromedial and posterolateral fiber bundles, with a visible inter-bundle cleft. 2. Ribbon-like phenotype: Presented as a flat, broad, sheet-like ligament with no clear macroscopic separation between fiber bundles. 3. Intermediate phenotype: Showed partial bundle differentiation, with some fiber grouping present but lacking complete cleft separation between the anteromedial and posterolateral portions. All classifications were independently evaluated by two observers, and any discrepancies between observers were resolved through joint re-evaluation. Two independent classification systems were adopted: one for ACL fiber-pattern (ribbon-like, two-bundle, intermediate), and another for bony footprint contour (oval, teardrop, irregular). The two intermediate-type specimens were not excluded but assigned to footprint-contour subgroups for coverage simulation.

### 2.5 Tunnel-to-footprint coverage simulation

The simulation of predicted femoral tunnel-to-footprint coverage was performed for perfectly circular tunnels with diameters of 8 mm, 9 mm, and 10 mm. In this simulation, each circular tunnel was positioned at the geometric centroid of the native ACL femoral footprint, representing a central placement. The overlap area between the circular tunnel and the irregular native footprint contour was calculated using ImageJ's pixel-based planar projected overlap method, rather than a simple area-to-area ratio of independent shapes. The coverage percentage was defined as: (overlapping area/native footprint area) × 100%. This centroid-aligned central placement represents an idealized scenario; deviations from central tunnel positioning in actual surgical procedures are likely to further diminish the actual footprint coverage.

### 2.6 Femoral footprint morphometric analysis

The femoral footprint was exposed and documented using a Canon EOS R5 camera equipped with a macro lens and a calibrated scale. Three-dimensional measurements included the maximum length (Lmax), maximum width (Wmax), and area, which were calculated using two methods: ImageJ planimetry and elliptical approximation (Area = π×Lmax/2×Wmax/2). Footprints were categorized as oval (length-to-width ratio of 1.5–2.5), teardrop (ratio>2.5), or irregular (ratio<1.5 or morphologically asymmetric). These cut-off values for the length-to-width ratio were predetermined for this study to differentiate between moderately elongated oval footprints, highly elongated teardrop-shaped footprints, and rounded or asymmetric irregular specimens. All measurements were conducted by two independent observers, with inter-observer reliability found to be excellent (ICC >0.90). The primary footprint-area values presented in Tables 1–3 were obtained from ImageJ manual planimetry (gold-standard manual tracing). The elliptical-approximation calculation was implemented for secondary comparative analysis only. We compared area outcomes from the two approaches; good agreement was observed between ImageJ planimetry and elliptical approximation.

**Table 1 Morphometric:** comparison between paired right and left knees. Mean Difference represents signed right-minus-left difference. 95% confidence intervals for mean differences were calculated for each paired comparison. P-values were obtained from paired-samples t-tests. Intra-class correlation coefficient (ICC, two-way random-effects, absolute-agreement model) was used to evaluate contralateral measurement consistency. *n* = 15 paired cadaveric knees.

Parameter	Right Knee (*n*=15)	Left Knee (*n*=15)	Mean Difference	*p*-value	ICC (95% CI)
ACL length (mm)	34.5 ± 2.8	34.9 ± 2.9	0.4 ± 2.1	0.63	0.96 (0.91–0.98)
ACL width (mm)	11.3 ± 1.7	11.5 ± 1.5	0.2 ± 1.4	0.71	0.94 (0.86–0.97)
Footprint area (mm²)	133.5 ± 19.8	134.1 ± 21.2	0.6 ± 12.4	0.91	0.94 (0.89–0.97)
Footprint length (mm)	17.2 ± 2.1	17.4 ± 2.3	0.2 ± 1.8	0.78	0.92 (0.84–0.96)
Footprint width (mm)	9.8 ± 1.4	9.7 ± 1.5	0.1 ± 1.2	0.85	0.91 (0.82–0.96)

### 2.7 Statistical analysis

Statistical analyses were conducted utilizing SPSS version 26.0. Continuous variables are reported as mean ± standard deviation (SD). Group comparisons were performed using either the t-test or the Mann–Whitney U test for continuous variables, while categorical variables were analyzed using the Chi-square test or Fisher's exact test. Correlations were evaluated using Pearson or Spearman correlation coefficients. Multivariate linear regression analysis was employed to identify independent predictors of footprint area. Statistical significance was determined at a *p*-value of less than 0.05 (two-tailed). Before collecting specimens, *a priori* sample size calculations were conducted for the primary endpoint, which was the sex-related difference in femoral footprint area. Drawing on previously published cadaver data, we anticipated an effect size of a 15% difference in area between sexes, with a standard deviation of 19 mm^2^ and an *α* level of 0.05 (two-sided). This calculation suggested that enrolling 14 specimens per group would provide at least 80% statistical power. This power analysis was conducted solely for the primary outcome, which was the sex-based comparison of footprint area. Subgroup analyses stratified by footprint morphology are presented descriptively, as the sample sizes for certain morphological categories were insufficient to achieve adequate statistical power. Inter-observer reliability was evaluated using a two-way random-effects, absolute-agreement intraclass correlation coefficient (ICC) model. Both observers were blinded to the donor's sex, knee side, and each other's measurement results during the morphometric assessment. The ICC values for each measured variable are presented with 95% confidence intervals (95% CI).

## 3 Results

### 3.1 Inter-observer reliability and side-to-side symmetry

Excellent inter-observer agreement was observed for all morphometric parameters using a two-way random-effects, absolute-agreement intraclass correlation coefficient (ICC) model. All observers were blinded to specimen sex, side, and each other's measurement results during data acquisition. The ICC values with 95% confidence intervals were as follows: footprint area (ICC = 0.94, 95% CI 0.89-0.97), ACL length (ICC = 0.96, 95% CI 0.91-0.98), ACL width (ICC = 0.94, 95% CI 0.86-0.97), footprint length (ICC = 0.92, 95% CI 0.84-0.96), and footprint width (ICC = 0.91, 95% CI 0.82-0.96). Bland-Altman limits of agreement demonstrated acceptable measurement consistency, satisfying standards for anatomical cadaver studies (Figure 2 and Table 1). For footprint area, the small signed mean difference of 0.6 ± 12.4 mm^2^ (right-left) occurs because positive and negative individual side-to-side values cancel one another: the near-zero mean demonstrates no systematic size bias between right and left knees, whereas the large standard deviation reflects wide inter-specimen scatter of individual side differences.

**Figure 2:**
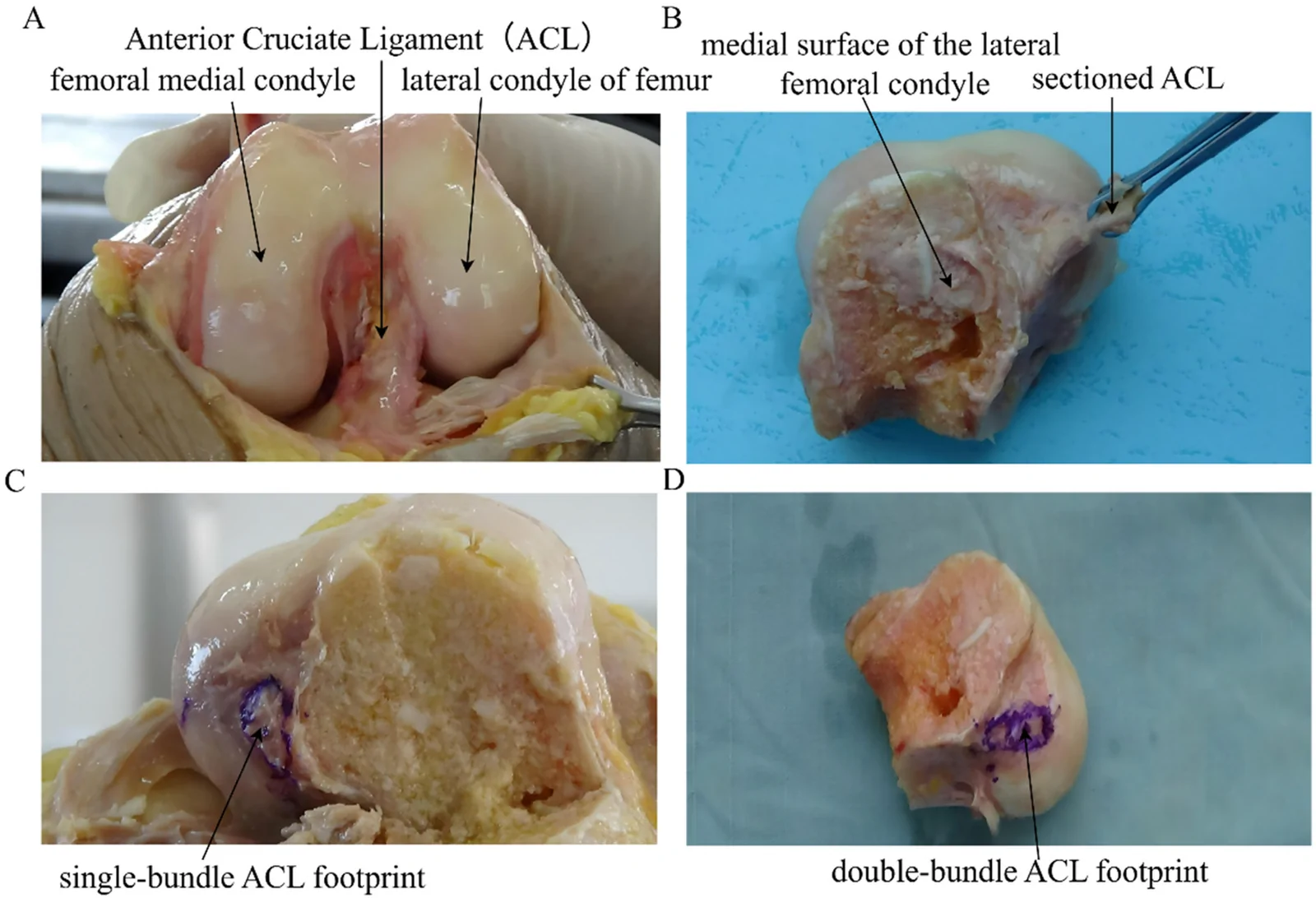
Illustrates the anatomical morphology of the ACL in a cadaveric knee: **(A,B)** a single strand of severed ACL, **(C)** the femoral attachment of a single strand of ACL, and **(D)** the partial attachment of a double strand ACL.

### 3.2 Femoral footprint morphology and dimensions

#### 3.2.1 Morphological distribution

Oval footprints were most prevalent (66.7%, *n* = 20), followed by teardrop (23.3%, *n* = 7) and irregular (10.0%, *n* = 3) configurations. Morphological type distribution did not differ significantly between sexes or sides. Dimensional Measurements (Tables 1, 2):Mean ACL length was 34.7 ± 2.8 mm, with no significant difference between sexes (males 34.6 ± 2.9 mm vs. females 34.8 ± 2.7 mm, *p* = 0.85) or sides (right 34.5 ± 2.8 mm vs. left 34.9 ± 2.9 mm, *p* = 0.63). Critical Finding—Footprint Area Variability: Mean femoral footprint area was 133.8 ± 20.4 mm^2^. Significant sex-based differences were observed: males demonstrated 16.3% larger footprint area compared to females (144.0 ± 19.1 mm^2^ vs. 123.6 ± 18.4 mm^2^, *p* < 0.01). This difference persisted when controlling for height and weight in multivariate analysis (*β*=15.2 mm^2^, 95% CI 6.8–23.6, *p* = 0.001), suggesting intrinsic anatomical dimorphism independent of body size. Side-to-side comparison revealed minimal asymmetry: right side 133.5 ± 19.8 mm^2^ vs. left side 134.1 ± 21.2 mm^2^ (*p* = 0.91), with mean absolute side difference 8.4 ± 6.2 mm^2^.

**Table 2:** Demographic and morphometric characteristics of the study cohort (*n* = 30 cadaveric knees from 15 donors). Continuous variables are presented as mea*n* ± standard deviation. Categorical morphological variables are shown as count (percentage). Between-group comparisons between male and female specimens were performed using independent-samples t-tests for continuous variables and *χ*^2^ test for categorical variables. Asterisk (*) indicates statistical significance at *p* < 0.05.

Characteristic	Overall (*n*=30)	Male (*n*=15)	Female (*n*=15)	*p*-value
Demographics				
Age at death (years)	62.4 ± 14.2	61.2 ± 15.1	63.6 ± 13.4	0.67
Height (cm)	165.4 ± 9.8	172.3 ± 6.8	158.4 ± 5.2	<0.001*
Weight (kg)	64.9 ± 12.1	71.2 ± 11.3	58.6 ± 9.7	0.003*
BMI (kg/m²)	23.6 ± 3.4	24.0 ± 3.8	23.2 ± 3.0	0.54
ACL Morphology				
Ribbon-like,n (%)	24 (80.0)	12 (80.0)	12 (80.0)	1
Two-bundle, *n* (%)	4 (13.3)	1 (6.7)	3 (20.0)	0.28
Intermediate, n (%)	2 (6.7)	2 (13.3)	0 (0)	0.14
ACL Dimensions				
Length (mm)	34.7 ± 2.8	34.6 ± 2.9	34.8 ± 2.7	0.85
Width (mm)	11.4 ± 1.6	12.3 ± 1.4	10.5 ± 1.5	0.002*
Footprint Characteristics				
Area (mm²)	133.8 ± 20.4	144.0 ± 19.1	123.6 ± 18.4	0.004*
Oval shape, n (%)	20 (66.7)	11 (73.3)	9 (60.0)	0.35
Teardrop shape, n (%)	7 (23.3)	3 (20.0)	4 (26.7)	0.63
Irregular shape, n (%)	3 (10.0)	1 (6.7)	2 (13.3)	0.54

### 3.3 The coverage gap: implications for standard techniques

Standard round tunnel techniques demonstrated substantial footprint under-coverage (Table 3). A conventional 9-mm tunnel provided mean coverage of only 47.5 ± 7.2%, leaving approximately half of the functional insertion site unaddressed. Coverage was significantly poorer in males than females for all tunnel sizes (*p* < 0.05), attributable to larger male footprint area. Even maximal 10-mm tunnels achieved <70% coverage in the majority of specimens. These findings highlight a critical anatomical mismatch in current surgical practice and provide quantitative rationale for footprint-matched tunnel designs.

**Table 3. Simulated:** footprint coverage of standard circular femoral tunnels of 8 mm, 9 mm, and 10 mm diameter. Mean area and percentage coverage are presented as mean ± standard deviation. Coverage percentages were calculated by geometric overlap simulation with the tunnel centered on the native ACL footprint. Asterisk (*) indicates statistically significant difference between male and female groups (*p* < 0.05).

Footprint Morphology	n	Mean Area (mm²)	8mm Tunnel Coverage (%)	9mm Tunnel Coverage (%)	10mm Tunnel Coverage (%)
Overall	30	133.8 ± 20.4	37.6 ± 5.7	47.5 ± 7.2	58.8 ± 8.9
By Sex					
Male	15	144.0 ± 19.1	35.0 ± 4.8*	44.2 ± 6.1*	54.8 ± 7.5*
Female	15	123.6 ± 18.4	40.2 ± 5.9*	50.8 ± 7.3*	62.8 ± 9.1*
By Morphology					
Oval	20	139.2 ± 18.6	36.2 ± 5.1	45.8 ± 6.4	56.7 ± 7.9
Teardrop	7	118.4 ± 15.2	42.6 ± 6.8	53.7 ± 8.6	66.5 ± 10.6
Irregular	3	128.6 ± 22.4	39.1 ± 6.8	49.4 ± 8.6	61.1 ± 10.6

## 4 Discussion

This study demonstrates that although ribbon-like anterior cruciate ligament (ACL) morphology is predominant, there is considerable inter-individual variability in the characteristics of the femoral footprint, which cannot be predicted using simple anthropometric measures. A strong correlation was observed between ACL width and footprint area (r = 0.72). This finding may have implications for preoperative phenotype characterization. Nevertheless, no correlation was detected with height or weight, which is inconsistent with conventional assumptions for graft sizing.

Our findings are consistent with the emerging paradigm of Precision Anatomic ACL Reconstruction (16). We propose a phenotype-guided algorithm: for oval large footprints (>140 mm^2^, representing 40% of the cohort), elliptical or rectangular tunnels (6 × 10 mm) with footprint-matched grafts may be considered; for small oval/teardrop configurations (<120 mm^2^, representing 30%), standard round tunnels may suffice. The area cut-offs of 120 mm^2^ and 140 mm^2^ were defined *a priori* based on the distribution of footprint-area values within the current cadaver cohort, aiming to stratify small, intermediate, and large-sized femoral footprints for hypothesis-generating purposes, without relying on external reference standards. True two-bundle phenotypes (13.3%) and intermediate phenotypes (6.7%, totaling 20%) may benefit from double-bundle reconstruction, along with irregular footprints (10%, *n* = 3). Given the limited sample size of this morphological subgroup, the use of patient-specific 3D-printed guides, based on anatomical features, may be considered. These approaches require further validation in larger cohorts and should be viewed as anatomically derived hypotheses pending future validation, rather than established clinical recommendations.

The minimal observed side-to-side asymmetry suggests that contralateral knee MRI could serve as a reference for preoperative planning when ipsilateral imaging is compromised. Additionally, sex-specific differences in footprint area (16.3% larger in males) persisted after controlling for body size, indicating intrinsic anatomical dimorphism that may inform sex-specific surgical planning rather than simple scaling based on patient height.

Recent biomechanical studies corroborate our recommendations, indicating that rectangular tunnel techniques exhibit superior restoration of rotational stability compared to conventional round tunnels (15, 17, 18). While emerging technologies including 3D-printed patient-specific navigation templates can improve bone-tunnel positioning accuracy and reduce intraoperative positioning time in complex anatomical ACL reconstruction (19), such techniques remain prospective and have not been validated in the current cadaver-based investigation. Post-operative tunnel-positioning can be reliably evaluated using 3D- CT or MRI (20). It is crucial to note that the percentage of femoral footprint coverage does not equate to the restoration of native anterior cruciate ligament (ACL) biomechanics. The outcomes of reconstruction are multifactorial, being influenced by variables such as graft tensioning, tunnel positioning, graft orientation, graft size, fixation technique, tibial footprint morphology, knee size, and rotational laxity. Adequate footprint coverage represents only one anatomical target and cannot independently ensure favorable results; thus, our findings should be interpreted with these clinical considerations in mind.

Another limitation of this study is the relatively advanced mean age of the cadaver cohort (62.4 ± 14.2 years). Age-related degenerative changes in the anterior cruciate ligament (ACL) and its femoral footprint may be present in older specimens, potentially altering the native morphology of the ligament and the dimensions of its footprint. Consequently, caution should be exercised when generalizing our morphometric findings to younger patient populations. This study is an exploratory morphometric analysis involving 30 cadaveric knees. Due to the limited sample size, the phenotype-guided algorithm proposed should be considered preliminary.

## Data Availability

The original contributions presented in the study are included in the article/Supplementary Material, further inquiries can be directed to the corresponding author.
